# Behaviour and sun exposure in holidaymakers alters skin microbiota composition and diversity

**DOI:** 10.3389/fragi.2023.1217635

**Published:** 2023-08-08

**Authors:** Thomas Willmott, Paul M. Campbell, Christopher E. M. Griffiths, Clare O’Connor, Michael Bell, Rachel E. B. Watson, Andrew J. McBain, Abigail K. Langton

**Affiliations:** ^1^ School of Health Sciences, The University of Manchester, Manchester, United Kingdom; ^2^ Centre for Dermatology Research, Manchester Academic Health Science Centre, The University of Manchester and Salford Royal NHS Foundation Trust, Manchester, United Kingdom; ^3^ NIHR Manchester Biomedical Research Centre, Manchester University NHS Foundation Trust, Manchester Academic Health Science Centre, Manchester, United Kingdom; ^4^ No7 Beauty Company, Walgreens Boots Alliance, Nottingham, United Kingdom; ^5^ A*STAR Skin Research Laboratory (A*SRL), Agency for Science, Technology and Research (A*STAR), Singapore, Singapore

**Keywords:** epidermal homeostasis, skin, skin microbiota, sun exposure behaviour, ultraviolet radiation

## Abstract

**Introduction:** The skin microbiota plays a crucial role in maintaining epidermal homeostasis. Ultraviolet radiation (UVR) and other environmental challenges can impact the skin microbiota through direct and indirect mechanisms. This study aimed to investigate the effects of sun exposure on the skin microbiota and its relationship with individual skin phototypes.

**Methods:** Healthy volunteers (*n* = 21 [4M, 17 F], mean age 33.2 years) holidayed in a sunny destination for a minimum of 7 days with swabs taken pre-holiday and up to 84 days post-holiday. Participant group was categorised by individual typology angle (ITA) classification and the composition of the skin microbiota was examined using 16S rRNA gene sequencing.

**Results:** In the entire cohort and at all time points, the major bacterial phyla were Actinobacteria, Proteobacteria and Firmicutes. There was a significant change in microbial beta diversity at day 28 post-holiday, compared to baseline, for all participants. However, when participants were segregated into three cohorts dependent on the degree of skin tanning response between baseline (pre-holiday) and immediately one-day post-holiday, there was a reduction in Proteobacteria in the sun-seeking participants 1 day after the holiday, which recovered over time.

**Discussion:** These findings suggest that sun exposure can affect the diversity and composition of the skin microbiota, which may have downstream effects on skin health.

## Introduction

High doses of ultraviolet radiation (UVR) are associated with acute and chronic reductions in skin health, including damage to DNA in skin cells, inflammation and photoageing (Lee et al., 2020; Raimondi et al., 2020). Chronic exposure to UVR is the most preventable risk factor for skin cancer, with the British Association of Dermatologists, the British Skin Foundation and the American Academy of Dermatology all recommending sun-protection practices for those especially with lightly-pigmented skin, including seeking shade, applying sunscreen, wearing protective clothing and avoiding tanning to reduce risk (American Academy of Dermatology, 2018; Centers for Disease Control and Prevention, 2018; British Skin Foundation, 2020). However, many holidaymakers intentionally expose themselves to high doses of UVR through sun-seeking behaviours and are therefore at increased risk of skin damage through this acute exposure. Sun-related behaviours vary widely between individuals, with often low engagement in sun-protection practices (Chen et al., 2019; Karlsson et al., 2021).

Human skin is home to a vast array of bacteria, fungi and viruses which compose the skin’s microbiota. These microbes play critical roles in skin homeostasis, including protection against invading pathogens, immune stimulation and the breaking down of host natural products (Scharschmidt and Fischbach, 2013; Belkaid and Segre, 2014; Grice, 2015). Microbial populations are organised within complex community structures and can influence the function of healthy skin (Prescott et al., 2017), with a disrupted microbiota negatively impacting the skin immune homeostatic network and contribute to systemic disease (Muir et al., 2016; Byrd et al., 2018; Lunjani et al., 2019; Suwarsa et al., 2021). As a dry and desiccating surface, human skin is largely unsuitable for most microbes to permanently reside (Vandegrift et al., 2017). However, there are niche populations which reside continuously on the skin, differing across bodily sites dependent on whether skin is sebaceous (oily), moist or dry (Byrd et al., 2018). Being open to the external environment, these communities are subject to both internal and external ecological pressures. One such major external pressure on the skin microbiota is UVR (Burns et al., 2019; Patra et al., 2020), but research on its effect on the skin microbiota *in vivo* is limited. It has, however, been shown that UVR can influence both the phylogenetic and genotypic composition, and the activity of these microbial communities (Rothschild, 1999; Patra et al., 2016; Burns et al., 2019), but subsequent downstream effects on host skin health are ambiguous (Souak et al., 2021). Some studies report positive effects of decreasing opportunistic pathogens (Krutmann, 2000) and increasing the production of UVR-absorbing porphyrins (Wang et al., 2012), whilst others show chronic inflammation and skin disease as a result of UVR-induced microbiota imbalances (Patra et al., 2018). The skin-resident microbiota has also demonstrated a role in protecting the organ against UVR damage via the release of tumour-necrosis factor α and interleukin-6 (Patra et al., 2019; Heidari et al., 2020). Furthermore, the cutaneous microbiota is considered a source of compounds with both direct and indirect photoprotective properties (Souak et al., 2021). As the skin microbiota is ubiquitous across the body and reaches deep down into the appendages, the disruption of microbial components and the formation of bacterial antigens are likely to activate the host immune system (Patra et al., 2016). It is therefore clear that much remains to be understood about how skin microbiota is influenced by UVR and how such changes may influence local and systemic immune health and host epidermal homeostasis.

To the best of our knowledge, the impact of sun-seeking, pro-tanning behaviours on skin health in relation to the microbiota of the skin has not been interrogated. The current study examines the effect of sun-seeking behaviours on epidermal melanin content and surveys the skin microbiota in holidaymakers. Participants went on a high sun exposure holiday for a minimum of 7 days and individual typology angle (ITA) classification (Del Bino and Bernerd, 2013) and microbiota composition was analysed prior to and following 1-, 28-, and 84-days post-holiday. The effects of UVR exposure on the skin microbial populations was assessed by quantifying microbial diversity and changes in the abundance of major skin bacterial taxa. This pilot study aimed to determine if sun-seeking behaviours in holiday makers were associated with changes in ITA status and shifts in the skin microbiota that is typically associated with disease.

## Materials and methods

### Study participants

Healthy, white Northern European volunteers (mean age ± SD: 33.6 ± 6.4 years; men, *n* = 4; women, *n* = 17) were recruited to the study (for further demographic information see Supplementary Table S1). Local ethical approval was obtained from The University of Manchester Research Ethics Committee (reference 16302). Written informed consent was obtained from the participants and the study adhered to Declaration of Helsinki principles. Prior to taking a holiday in a sunny destination (minimum of 7 days duration), volunteers had skin swabs taken from their extensor forearm (d0) and upon their return skin swabs were repeated at d1, d28 and d84.

### Colorimetric measurements of skin and ITA determination

A Chroma Meter (CR-400; Konica Minolta, Warrington, United Kingdom) was used to measure the L* and b* parameters of the standard CIE L*a*b* colour space on the buttock and extensor forearm before (d0) and immediately after their holiday (d1) in order to assess whether a tanning response had occurred. Chroma Meter readings were converted to ITA values using the formula ITA = (arctan (L*-50)/b*) x 180/ π . ITA values allow skin colour types to be classified into six different groups as previously described (Del Bino and Bernerd, 2013): very light: > 55°; light: 41°–55°; intermediate: 28°–41°; tan: 10°–28°; brown: −30°–10°; dark: < −30.

### 16S rRNA gene sequencing and bioinformatics

Skin swabs, PCR water as a negative control and PCR water with a colony of *Staphylococcus aureus* (Newman strain) as a positive control were employed in the DNeasy PowerSoil Kit (Qiagen, Hilden, Germany) following the manufacturer’s instructions to isolate the microbial DNA. Subsequent amplification was achieved through 16S rRNA gene Polymerase Chain Reaction (PCR) with Illumina (San Diego, California, United States) adapted 515F/806R primers (Caporaso et al., 2012) and NEBNext^®^ High-Fidelity 2X PCR Master Mix (New England Biolabs; Ipswich, Massachusetts, United States) at 98°C (2 min) followed by 25 cycles of 95°C (20 s), 62°C (15 s), 70°C (30 s) and a final elongation step of 72°C (5 min), which have been previously validated in oral microbiome studies (Demmitt et al., 2017; Rullo et al., 2020; Su et al., 2020; Granato et al., 2021). The amplified 16S rRNA gene was purified using a QIAquick purification kit (Qiagen) and amplicons were confirmed by gel electrophoresis.

Sequencing of the 16S rRNA gene V4 amplicons was performed on the Illumina MiSeq platform (Illumina Inc.; Cambridge, United Kingdom). Raw sequence data were imported into the quantitative insights into microbial ecology (QIIME) version 2 (2020.2) (Caporaso et al., 2010; Bolyen et al., 2019). Sequences were de-replicated, similarity clustered, analysed for chimaeras, demultiplexed and quality filtered using the d2-demux plug-in followed by denoising with DADA2 (q2-dada2) (Callahan et al., 2016). Amplicon sequence variants (ASVs) were aligned via the q2-alignment plug-in and taxonomy assigned to the ASVs using the q2-feature-classifier (Bokulich et al., 2018) against the Greengenes (v13.8) 97% ASVs reference sequences (McDonald et al., 2012) for the generation of BIOM tables comprising the sample metadata. ASVs present in the negative controls were subtracted from the remaining samples. Following importation of data using the qiime2R package (Bisanz, 2018), analysis was performed using the Phyloseq package (McMurdie and Holmes, 2013), converting data into relative abundance, ggPlot2 (Wickham, 2016) was applied to generate figures in R version 3.6.2 (Team, 2022). Data is available from: https://www.ncbi.nlm.nih.gov/bioproject/987584; accession number: PRJNA987584.

## Results

**FIGURE 1:**
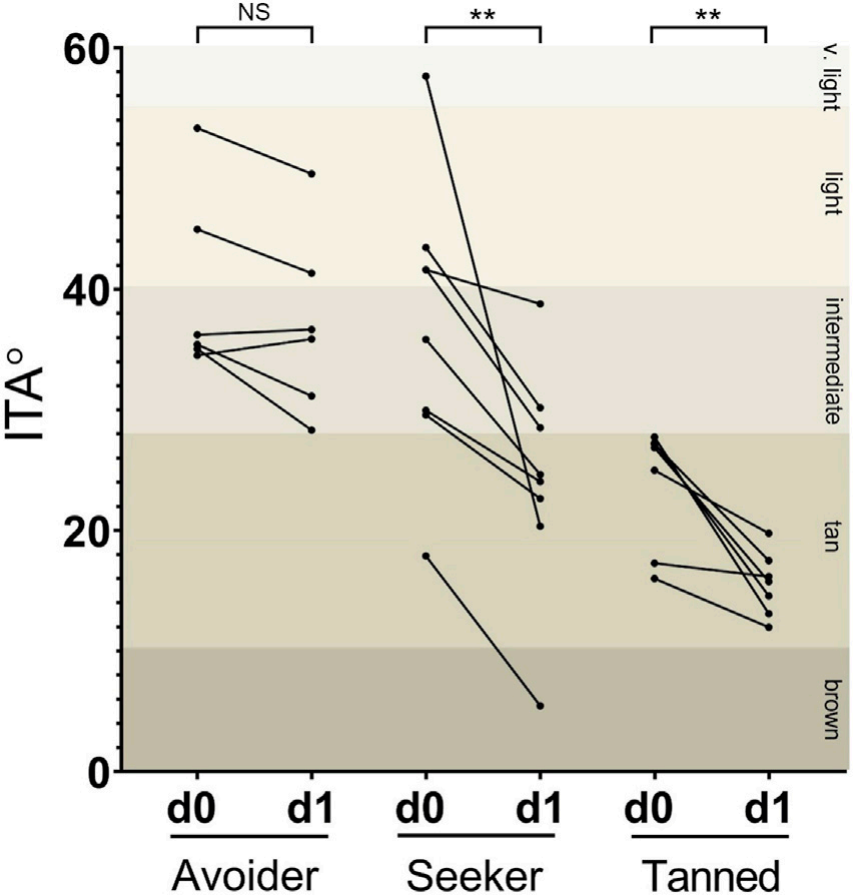
Participants could be segregated by degree of change in epidermal melanin following sun exposure on holiday. In total, *n* = 8 volunteers demonstrated a change in ITA classification following their holiday (“seeker” group); *n* = 7 had tanned skin pre-holiday and returned from holiday with tanned skin (“tanned” group); and *n* = 6 volunteers had light or intermediate skin pre-holiday and remained in these same classifications upon their return (“avoider” group). d0 = pre-holiday; d1 = 1-day post-holiday. ** *p* < 0.01.

A total of 21 participants were successfully recruited to the study and prior to taking a holiday in a sunny destination, volunteers had Chroma Meter readings taken from their buttock and extensor forearm (d0) and upon their return immediately post-holiday (d1). As expected, prior to taking their holiday the baseline skin colour of each participant at sun-protected buttock was lighter than at their sun-exposed extensor forearm. However, when skin colour was assessed on the extensor forearm immediately after their holiday and compared to their pre-holiday readings at the same anatomical site, it was apparent that the participants could be stratified into three groups that displayed different sun exposure behaviours. In total, *n* = 8 participants were deemed sun-seekers due to a change in their ITA classification from a lighter to a darker category following their sunny holiday (“seeker” group); *n* = 7 participants had tanned skin pre-holiday and returned from holiday with tanned skin (“tanned” group); whilst *n* = 6 participants were deemed sun-avoiders due to having light or intermediate ITA classifications pre-holiday and remained in these same classifications upon their return (“avoider” group; Figure 1).

**FIGURE 2:**
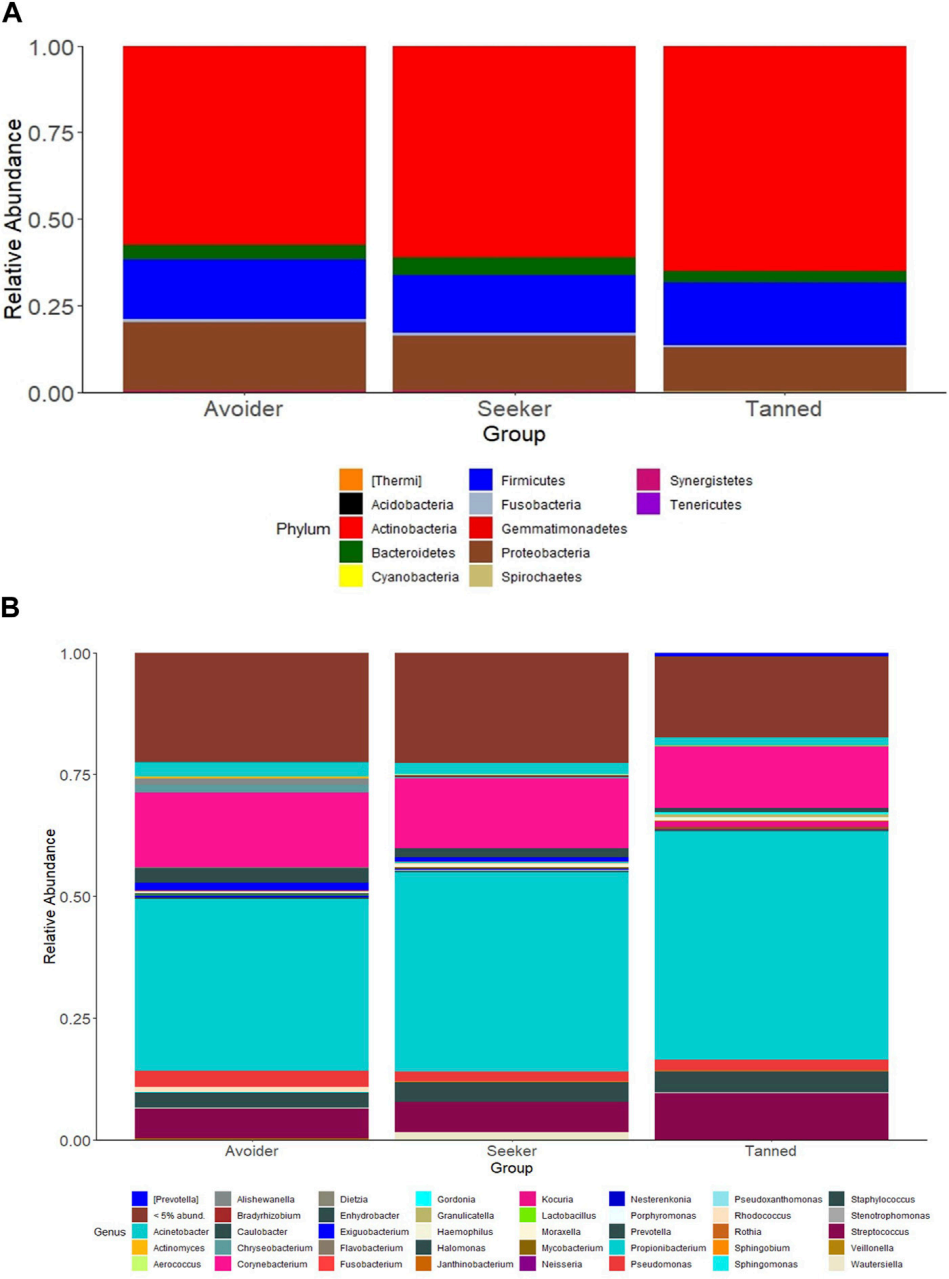
Relative abundances of bacterial taxa in the skin microbiome compared between “avoiders”, “seekers” and “tanned” individuals cross-sectionally at all timepoints. Distribution of skin bacterial taxa are presented at the **(A)** phylum and **(B)** genus level.

**FIGURE 3:**
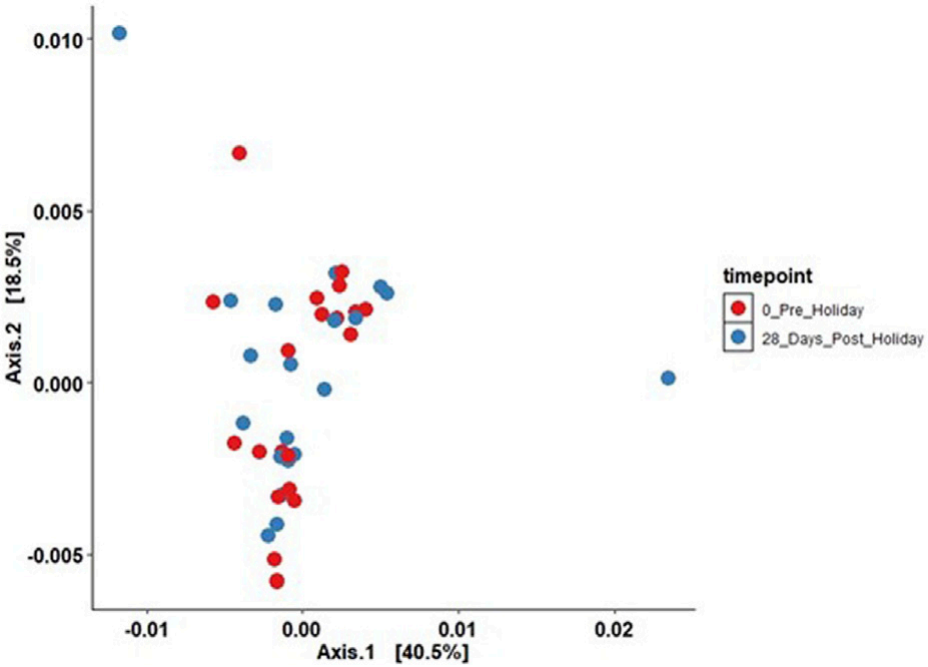
Principal components analysis plotting beta diversity (*p* = 0.03) between all participants between the pre-holiday (red) and 28-day post-holiday (blue) time points (unweighted UniFrac analysis; ADONIS analysis of variance using distance matrices).

The effects of the sunny holiday on the skin microbiome was first assessed by examining the bacterial communities on the surface of the skin at different taxonomic levels. Actinobacteria, Proteobacteria and Firmicutes were the topmost abundant phyla, comprising 94.1% of all sequences, across all individuals and time-points both pre- and post-holiday. The remaining 5.9% consisted of other detected phyla, including Bacteroidetes, Cyanobacteria and unassigned taxa (Figure 2A). At the genus level, *Propionibacterium*, *Corynebacterium* and *Streptococcus* were the most abundant genera, comprising 51.4% of the sequences respectively across all individuals and time-points both pre- and post-holiday (Figure 2B). There was no significant difference in the cross-sectional relative abundance of these phyla or genera between participants when all time points, both pre- and post-holiday, were combined (*p* > 0.05; Supplementary Table S2). Beta diversity is an ecological measure of similarity between multiple communities, capturing changes in community composition between categorically different samples (Wagner et al., 2018). Beta diversity is calculated using unweighted UniFrac, which measures the distance between two bacterial communities by calculating the fraction of the branch length in a phylogenetic tree that leads to descendants in either but not both of the two communities (Lozupone et al., 2007). Microbiome data is presented in a PCoA, in which points that are closer together represent microbial communities that are more similar in sequence composition (Goodrich et al., 2014). Compared to pre-holiday, there was a significant change in beta diversity at 28 days post-holiday when comparing all participants regardless of skin groups (*p* = 0.03, Figure 3; Supplementary Table S3).

**FIGURE 4:**
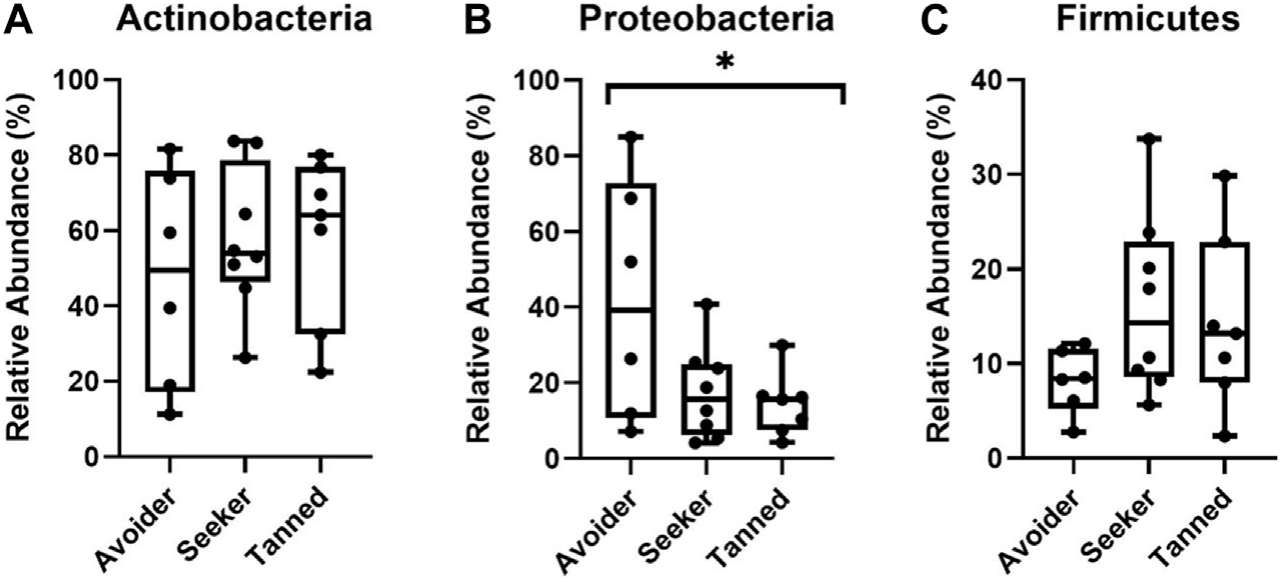
Relative abundance of the phyla **(A)** Actinobacteria **(B)** Proteobacteria and **(C)** Firmicutes 1 day post-holiday. There was significantly less Proteobacteria in the seeker and tanned participants (* = *p* < 0.05).

Using these participant groupings, the relative abundance of the three largest detected phyla, Actinobacteria, Proteobacteria and Firmicutes, were next examined at d1, d28 and d84 post-holiday. Upon the immediate return from holiday (d1), there was significantly less Proteobacteria in the “seeker” and “tanned” groups compared to the “avoider group” (*p* < 0.05) but no significant difference in the relative abundance of Actinobacteria and Firmicutes (*p* > 0.05; Figure 4). Furthermore, by d28 and d84 post-holiday timepoints there were no significant differences in any of the major phyla or genera between groups (*p* > 0.05; Supplementary Figure S1).

## Discussion

This study examined the effects of various behaviours during a high sun exposure holiday and how these influenced the composition and diversity of the skin microbiota. These data provide evidence that altered skin microbiome composition is associated with sun-seeking behaviour over a minimum seven-day period. Participants could be segregated by a change in ITA classification over the holiday period, with two groups of participants showing no change in classification (those who were either already tanned or were of lighter skin classifications prior to holiday sun exposure), whilst others had large degrees of change. The development of a tan was significantly associated with lower Proteobacteria relative abundance immediately post-holiday, with community structure recovering by 28- and 84-days post-holiday. To our knowledge, this is the first study to sample and analyse the changes in skin microbiota following a ‘real-life’ holiday setting, and to stratify the results based on changes in epidermal melanin levels as a measure of participant behaviours in response to the sun.

As sun exposure is the main modifiable risk factor for skin cancer, the relationship between sun exposure habitats and skin health has been previously examined. Sun-seeking behaviours of holidaymakers has been previously associated with adverse skin health (O'Riordan et al., 2008; Petersen et al., 2013b), with just 1 week sun exposure inducing the expression of photoageing biomarkers (Lesiak et al., 2016) and skin cancer associated with harmful sun-seeking travelling behaviours (Karlsson et al., 2021). A study of Danish holiday makers showed that just a 6 day holiday provided 43% of the annual UVR cumulative exposure for an indoor Danish worker (Petersen et al., 2013a). Two previous studies have shown that fair skinned individuals have safer sun exposure behaviours, such as wearing protective clothing and staying in the shade, than dark skinned individuals (Yan et al., 2015; Pettigrew et al., 2016), which was contradicted by Suppa *et al.* (Suppa et al., 2013). A study by Falk *et al.* showed that those with a personal history of skin cancer exhibited some avoidance behaviours (Falk et al., 2013). Therefore, the behaviour of holiday makers has a major impact on their personal UVR exposure, with sun-seeking, pro-tanning attitudes possibly increasing the risk of skin cancer (Diffey, 2008).

Differences in sun exposure behaviour as observed in this study are likely influenced by both social pressures and cultural beliefs. There are the social and group pressures that associate the tanned look with health and wellbeing (Bränström et al., 2004; Gambla et al., 2017). It has also been shown in animal models that β-endorphins, which block pain signalling and produce a feeling of pleasure, are synthesised in the skin and are elevated in the plasma following low-dose UVR exposure (Kourosh et al., 2010). Being male, of white Northern European ancestry and having sun sensitive skin, in addition to participating in water and non-water sports, sunbathing and vacations have also been associated with skin damage (Bowers et al., 2021). Winter holidays, such as skiing, have also shown to provide substantial UVR doses to holiday makers (Petersen et al., 2015). It is therefore important in the future to raise awareness to holidaymakers of the risk factors associated with sun seeking behaviours The underlying psychological reasons for intentional exposure to excessive UVR may be critical in reducing the global incidence of skin cancer.

In this study, bacterial communities could be grouped by similarity in these participant groups, despite there being no significant differences in the individual major bacterial taxa between these groups when comparing all time points combined. The bacterial communities on the surface of the skin are critical in epidermal homeostasis, with roles in skin barrier function, homeostasis and cutaneous immunity (Belkaid and Tamoutounour, 2016; Boxberger et al., 2021). Ultraviolet radiation is known to directly damage skin lipids, proteins and DNA associated with carcinogenesis (Santos et al., 2013; Lee et al., 2020). The effect of UVR exposure on the diversity and composition of the skin microbiota has been documented (Faergemann and Larkö, 1987; Burns et al., 2019). However, to the best of our knowledge, how these UVR-induced microbiome changes affect skin health has not been examined. We have demonstrated that the development of a tan is significantly associated with lower Proteobacteria relative abundance immediately post-holiday (d1), with community structure recovering by 28 days post-holiday. This is important because it indicates that UV exposure on holiday has an acute effect on the skin microbiome but recovery is relatively rapid once the person returns to a less sunny climate. It has been shown that Proteobacteria dominate the skin microbiota and therefore it is not surprising that there would be rapid recovery of the microbiome to re-establish homoeostasis (Grice et al., 2008). Alterations in the human microbiome diversity has been linked to disease states. Typically, an increase or decrease in microbial diversity is associated with disease (Lloyd-Price et al., 2016; Byrd et al., 2018), with reduced bacterial alpha diversity linked to obesity and IBD (Andoh and Nishida, 2022; Manos, 2022; van der Vossen et al., 2022) while increased diversity in the vaginal microbiome is associated with bacterial vaginosis (Onderdonk et al., 2016; Abou Chacra et al., 2022). The use of systemic antibiotics for the treatment of acne vulgaris resulted in reduced skin microbial diversity (Chien et al., 2019), whilst diversity is altered in the skin microbiota of psoriasis patients (Chang et al., 2018). A decrease in skin bacterial richness has been previously associated with atopic dermatitis (Hanski et al., 2012). Therefore, like other human microbiota, the richness and diversity of bacterial species on the skin is associated with disease. It is therefore plausible that loss of bacterial diversity observed with increased facultative tanning may also impact skin health.

Skin microbial communities could be segregated by beta diversity (i.e., community similarity). Despite there being no significant differences in the major bacterial genera by tanning response, there was decreased Proteobacteria in those in the sun-seeking (“seeker” and “tanned”) groups 1 day post-holiday at the phylum level. How bacteria react and survive following UVR exposure can vary greatly. It has been suggested UVR affects bacterial groups differently, with Gram-positive organisms having greater protection from damage than Gram-negative organisms (Matallana-Surget et al., 2008). Proteobacteria is a large phylum of Gram-negative bacteria commonly isolated from healthy human skin (Cosseau et al., 2016; Egert and Simmering, 2016). A disturbed Proteobacteria microbiota has been previously associated with psoriasis (Alekseyenko et al., 2013), eczema (Zeeuwen et al., 2017) and diabetic foot ulcers (Gardner et al., 2013), with greater Proteobacteria diversity correlated with T-helper_1_, interleukin-10 and anti-inflammatory immune responses that were protective against allergic inflammation (Hanski et al., 2012; Fyhrquist et al., 2014). Thus, a Proteobacteria imbalance may suggest decreased skin health in those sun-seeking individuals. Varied tolerances to UVR within the Gram-negative bacteria have been reported (Oppezzo et al., 2011) and the results of this current study may be attributed to reduced coping mechanisms and tolerance to UVR within this group of bacteria which is related to poor skin health. Despite these differences 1 day post-holiday, there were no differences 28- and 86-days post-holiday, suggesting communities did recover following the removal of the high UVR exposure on the holiday.

There have been limited studies on the effect of sun exposure on the skin microbiota and - to the best of our knowledge–no studies which included how an individual’s behaviour influences UVR-associated microbiota shifts and how this may relate to skin health. A strength of this study is the multi-faceted study design. Objective measurement of epidermal melanin enabled the direct characterisation of microbial responses to UVR exposure. One limitation of the current study was the small number of participants (*n* = 21), which when subdivided by changes in epidermal melanin resulted in only between six and eight participants per group. Future studies should aim to increase the number of participants. In addition, as >80% of our volunteers were female, it would be interesting to understand if sex is associated with alterations in skin microbiota composition and diversity. Furthermore, due to limitations of presenting microbiota as compositional relative abundances, often leading to the misinterpretation of the biological relevance of shifts due to over or underrepresenting taxon abundances, future analyses should target the microbiota using quantitative and semi-quantitative techniques such as qPCR (Knight et al., 2018; Lin et al., 2019; Jian et al., 2020). It would have also been useful to measure individual UVR exposures over their holiday instead of using tanning responses as an indirect measure.

We conclude that in this mixed-sex study population of “real-life” holidaymakers, sun exposure resulting in a tanning response leads to an acute reduction in Proteobacteria abundance and decreased skin microbiota diversity which recovers by d28. Future studies might examine why members of the phyla Proteobacteria are particularly sensitive to UVR and how this change in diversity impacts host skin health in the longer term. Furthermore, it should be examined if topical application of Proteobacteria before sun exposure would have a protective, beneficial effect, limiting the alterations in skin microbiota diversity observed in this present study. These findings could potentially provide insight into UVR-induced inflammation and damage and into other skin disorders associated with shifts in the microbiota. Increased knowledge is required to thoroughly understand the protective nature of skin microbiota and how alterations contribute to skin health and disease. Ultimately this will inform the design of new interventions, such as topical pre- and probiotics (Patra et al., 2020; Souak et al., 2021), that could promote host dermatological health in response to sun exposure.

## Data Availability

The datasets presented in this study can be found in online repositories. The names of the repository/repositories and accession number(s) can be found below: https://www.ncbi.nlm.nih.gov/, PRJNA987584.

## Abbreviations

ITA, individual typology angle; PCR, polymerase chain reaction; UVR, ultraviolet radiation.
