# Supplementary material for: Behaviour and sun exposure in holidaymakers alters skin microbiota composition and diversity
Source: Front Aging. 2023 Aug 8;4:1217635. doi: 10.3389/fragi.2023.1217635 (PMC10442491; doi:10.3389/fragi.2023.1217635)
Supplement: Supplementary file 1 [file Table1.DOCX]

| **Volunteer** | **Group** | **Age** | **Sex** | **Destination** | **Duration (days)** | **Sunscreen Used?** |
| --- | --- | --- | --- | --- | --- | --- |
| 1 | Avoider | 39 | F | Spain | 7 | Yes |
| 2 | Avoider | 28 | F | Turkey | 10 | Yes |
| 3 | Avoider | 30 | F | Vietnam | 14 | Yes |
| 4 | Avoider | 41 | F | Portugal | 10 | Yes |
| 5 | Avoider | 40 | F | Greece | 7 | Yes |
| 6 | Avoider | 38 | M | Spain | 7 | Yes |
| 7 | Seeker | 31 | F | Turkey | 21 | Yes |
| 8 | Seeker | 40 | F | Spain | 7 | Yes |
| 9 | Seeker | 25 | F | Spain | 7 | Yes |
| 10 | Seeker | 27 | F | Gran Canaria | 7 | Yes |
| 11 | Seeker | 25 | M | Zante | 10 | Yes |
| 12 | Seeker | 38 | F | Spain | 7 | Yes |
| 13 | Seeker | 32 | F | Portugal | 7 | Yes |
| 14 | Seeker | 33 | F | Malta | 14 | Yes |
| 15 | Tanned | 34 | F | Ibiza | 7 | Yes |
| 16 | Tanned | 41 | F | Turkey | 10 | Yes |
| 17 | Tanned | 39 | F | Turkey | 14 | Yes |
| 18 | Tanned | 33 | M | Turkey | 14 | Yes |
| 19 | Tanned | 40 | M | Gran Canaria | 7 | Yes |
| 20 | Tanned | 20 | F | Tenerife | 7 | Yes |
| 21 | Tanned | 27 | F | Portugal | 10 | Yes |

**Supplementary Table 1. Demographic information**
