# Supplementary material for: Behaviour and sun exposure in holidaymakers alters skin microbiota composition and diversity
Source: Front Aging. 2023 Aug 8;4:1217635. doi: 10.3389/fragi.2023.1217635 (PMC10442491; doi:10.3389/fragi.2023.1217635)
Supplement: Supplementary file 2 [file Table2.docx]

**Supplementary Table 2. Alpha Diversity (Longitudinal)**

|  | Alpha Pairwise Differences | | | | |
| --- | --- | --- | --- | --- | --- |
|  |  | Pre vs 1 (p.adj) | Pre vs 7 (p.adj) | Pre vs 28 (p.adj) | Pre vs 84 (p.adj) |
| Observed ASVs | Avoider | 0.34 | 0.92 | 0.35 | 0.69 |
|  | Seeker | 0.67 | 0.69 | 0.35 | 0.35 |
|  | Tanned | 0.34 | 0.69 | 0.35 | 0.69 |
|  | [Pairwise] | 0.14 | 0.68 | 0.25 | 0.50 |
| Shannon's DI | Avoider | 0.74 | 0.46 | 0.75 | 0.13 |
|  | Seeker | 0.74 | 0.05 | 0.35 | 0.37 |
|  | Tanned | 0.74 | 0.46 | 0.47 | 0.89 |
|  | [Pairwise] | 0.41 | 0.09 | 0.09 | 0.08 |
| Faith's PD | Avoider | 0.50 | 0.92 | 0.50 | 0.89 |
|  | Seeker | 0.50 | 0.60 | 0.35 | 0.14 |
|  | Tanned | 0.50 | 0.60 | 0.50 | 0.89 |
|  | [Pairwise] | 0.41 | 0.82 | 0.26 | 0.62 |
| Pielou's Evenness | Avoider | 0.50 | 0.92 | 0.92 | 0.13 |
|  | Seeker | 0.50 | 0.08 | 0.22 | 0.37 |
|  | Tanned | 0.50 | 0.69 | 0.36 | 0.89 |
|  | [Pairwise] | 0.42 | 0.16 | 0.10 | 0.06 |

**Supplementary Table 3. Beta Diversity (Longitudinal)**

|  | Beta Pairwise Distances | | | | |
| --- | --- | --- | --- | --- | --- |
|  |  | Pre vs 1  **(p)** | Pre vs 7  **(p)** | Pre vs 28  **(p)** | Pre vs 84  **(p)** |
| Jaccard | Overall | 0.15 | 0.50 | 0.09 | 0.08 |
|  | Avoider vs Seeker | - | - | - | - |
|  | Avoider vs Tanned | - | - | - | - |
|  | Seeker vs Tanned | - | - | - | - |
| Bray-Curtis | Overall | 0.51 | 0.60 | 0.83 | 0.27 |
|  | Avoider vs Seeker | - | - | - | - |
|  | Avoider vs Tanned | - | - | - | - |
|  | Seeker vs Tanned | - | - | - | - |
| Unweighted UniFrac | Overall | 0.18 | 0.14 | **0.03** | 0.07 |
|  | Avoider vs Seeker | - | - | **0.02** | - |
|  | Avoider vs Tanned | - | - | 0.43 | - |
|  | Seeker vs Tanned | - | - | 0.20 | - |
| Weighted UniFrac | Overall | 0.71 | 0.24 | 0.42 | 0.60 |
|  | Avoider vs Seeker | - | - | - | - |
|  | Avoider vs Tanned | - | - | - | - |
|  | Seeker vs Tanned | - | - | - | - |
