# Supplementary material for: Behaviour and sun exposure in holidaymakers alters skin microbiota composition and diversity
Source: Front Aging. 2023 Aug 8;4:1217635. doi: 10.3389/fragi.2023.1217635 (PMC10442491; doi:10.3389/fragi.2023.1217635)
Supplement: Supplementary file 3 [file DataSheet1.docx]

**Supplementary Figure 1.**

The relative abundance of Proteobacteria for each group across the four time points.
